# Diagnostic concordance between BioFire® FilmArray® Pneumonia Panel and culture in patients with COVID-19 pneumonia admitted to intensive care units: the experience of the third wave in eight hospitals in Colombia

**DOI:** 10.1186/s13054-022-04006-z

**Published:** 2022-05-09

**Authors:** Francisco José Molina, Luz Elena Botero, Juan Pablo Isaza, Luz Elena Cano, Lucelly López, Leidy Tamayo, Antoni Torres

**Affiliations:** 1 Escuela de Ciencias de La Salud, Facultad de Medicina, Universidad Pontificia Bolivariana, Medellín, Colombia; 2 Intensive Care Unit, Clínica Universitaria Bolivariana, Universidad Pontificia Bolivariana, Medellín, Colombia; 3 Corporación Para Investigaciones Biológicas, Medellín, Colombia; 4 Medicine (Pulmonology), University of Barcelona, Barcelona, Spain; 5 The Respiratory and Intensive Care Unit, Hospital Clinic of Barcelona, Barcelona, Spain

**Keywords:** COVID-19, FilmArray, Bacterial coinfection, Bacterial pneumonia, Intensive care units

## Abstract

**Background:**

The detection of coinfections is important to initiate appropriate antimicrobial therapy. Molecular diagnostic testing identifies pathogens at a greater rate than conventional microbiology. We assessed both bacterial coinfections identified via culture or the BioFire® FilmArray® Pneumonia Panel (FA-PNEU) in patients infected with SARS-CoV-2 in the ICU and the concordance between these techniques.

**Methods:**

This was a prospective study of patients with SARS-CoV-2 who were hospitalized for no more than 48 h and on mechanical ventilation for no longer than 24 h in 8 ICUs in Medellín, Colombia. We studied mini-bronchoalveolar lavage or endotracheal aspirate samples processed via conventional culture and the FA-PNEU. Coinfection was defined as the identification of a respiratory pathogen using the FA-PNEU or cultures. Serum samples of leukocytes, C-reactive protein, and procalcitonin were taken on the first day of intubation. We analyzed the empirical antibiotics and the changes in antibiotic management according to the results of the FA-PNEUM and cultures.

**Results:**

Of 110 patients whose samples underwent both methods, FA-PNEU- and culture-positive samples comprised 24.54% versus 17.27%, respectively. Eighteen samples were positive in both techniques, 82 were negative, 1 was culture-positive with a negative FA-PNEU result, and 9 were FA-PNEU-positive with negative culture. The two bacteria most frequently detected by the FA-PNEU were *Staphylococcus aureus* (37.5%) and *Streptococcus agalactiae* (20%), and those detected by culture were *Staphylococcus aureus* (34.78%) and *Klebsiella pneumoniae* (26.08%). The overall concordance was 90.1%, and when stratified by microorganism, it was between 92.7 and 100%. The positive predictive value (PPV) was between 50 and 100% and were lower for *Enterobacter cloacae* and *Staphylococcus aureus*. The negative predictive value (NPV) was high (between 99.1 and 100%); MecA/C/MREJ had a specificity of 94.55% and an NPV of 100%. The inflammatory response tests showed no significant differences between patients whose samples were positive and negative for both techniques. Sixty-one patients (55.45%) received at least one dose of empirical antibiotics.

**Conclusions:**

The overall concordance was 90.1%, and it was between 92.7% and 100% when stratified by microorganisms. The positive predictive value was between 50 and 100%, with a very high NPV.

## Introduction

Critically ill patients with COVID-19 have a high mortality rate (38.4%), mainly related to advanced age, the severity of illness on intensive care unit (ICU) admission, vasopressor support, and renal replacement therapy [1]. A meta-analysis reported an incidence of 7% of hospitalized COVID-19 patients with bacterial coinfection; this proportion increased to 14% in studies that only included patients who required ICU admission, almost universally from studies utilizing culture-based methods [2]. In the most recent study, Langford et al. found that the prevalence of overall respiratory and/or bloodstream bacterial coinfection in patients with COVID-19 was 4.4% (95% CI 3.0–6.4%) in hospitalized patients and 15.4% (95% CI 10.5–22.0%) in intensive care patients; meta-regression revealed potential risk factors for infection, including ICU setting and mechanical ventilation [3]. Concern over bacterial coinfections has led to significant antimicrobial use in up to 80% of critically ill COVID-19 patients [4], although early administration of antibiotics does not impact mortality in these subjects [5]; therefore, strategies must be established for improving antimicrobial stewardship in COVID-19.

Molecular tests provide a more rapid turnaround time, early isolations, semiquantitative results for many pathogens, and antibiotic resistance markers, thus improving antimicrobial stewardship. The BioFire® FilmArray® Pneumonia Panel (FA-PNEU) detects severe pneumonia pathogens at a greater rate than conventional microbiology tests. In a substudy of the PROGRESS trial, sputum samples of 90 patients with sepsis and lower respiratory tract infection (LRTI) were retrospectively analyzed; the FA-PNEU detection rate was 72.2% compared to 10% based on conventional microbiology (*p* < 0.001) [6]. Few data are available on the use of molecular techniques for the identification of bacterial pathogens in the respiratory tract of critically ill COVID-19 patients [7–10].

There are several limitations of previous investigations: Few studies of pulmonary coinfection have been carried out only in ventilated patients in the ICU who have not been hospitalized for more than 48 h that analyze the concordance of cultures with molecular assays and prompt changes in antibiotics according to the results of these tests. In this prospective multicenter study, we assessed bacterial coinfections based on cultures or the FA-PNEU in the first lower respiratory tract sample taken from SARS-CoV-2 patients hospitalized in an ICU. We analyzed the concordance between conventional cultures and the FA-PNEU.

## Materials and methods

### Study setting

**Fig. 1:**
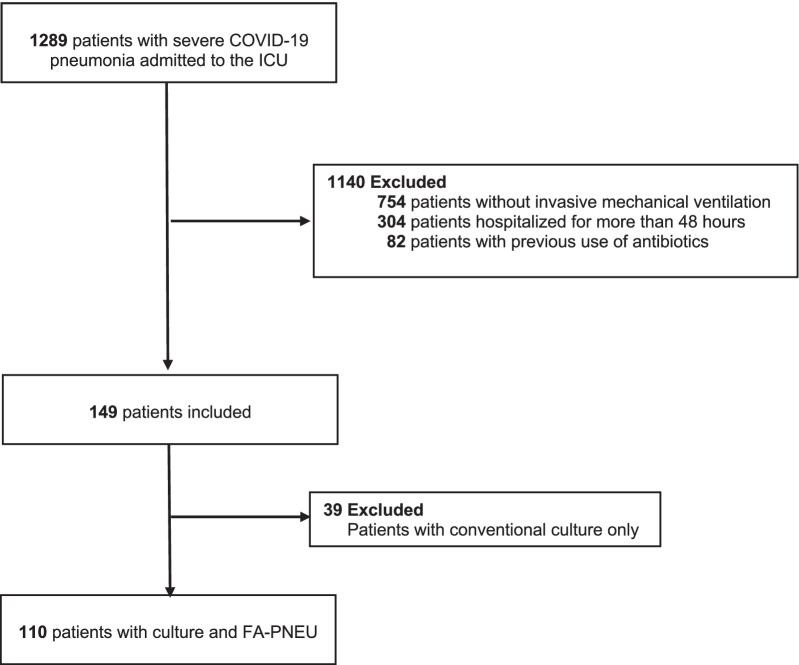
Study flowchart. ICU, intensive care unit; FA-PNEU, BioFire® FilmArray® Pneumonia Panel

We performed a prospective study of 149 patients with laboratory-confirmed SARS-CoV-2 infection who were hospitalized from March 1 to July 30, 2021, at eight ICUs in Medellín, Colombia. Inclusion criteria included patients over 18 years of age with severe COVID-19 infection consecutively admitted to the ICU according to NIH criteria [11] on mechanical ventilation. To meet coinfection criteria, patients could not have been hospitalized for more than 48 h at the time of LRT sampling. Patients who had received any dose of empiric antimicrobial therapy were excluded (Fig. 1). However, the treating physicians were free to initiate antibiotics after taking the respiratory samples and to make changes in the antimicrobial therapy according to the results of the FA-PNEUM and the cultures.

Sample size was calculated to estimate the sensitivity and specificity for *Staphylococcus aureus* based on the article by Kolenda et al., where the expected sensitivity for this same microorganism was 99.9% and the specificity was 93.5%, with an absolute precision of 5%, obtaining a necessary sample of 96 patients [8].

The respiratory therapists of each ICU took samples from the lower respiratory tract on the first day of intubation with mini-bronchoalveolar lavage fluid [mini-BAL] or endotracheal aspirate [ETA] for conventional culturing and the FA-PNEU. Of the total volume of samples, 5–10 ml (mL) was distributed for FA-PNEU testing, 5–10 mL for conventional culturing, and another 5–10 mL stored for lung microbiome analysis with the extraction of deoxyribonucleic acid (DNA) and ribonucleic acid (RNA) for metagenomic and metatranscriptomic sample sequencing. (The results of these tests are part of another study.) According to microorganism identification through the FA-PNEU, antibiotics were initiated, considering the pathogen and resistance profile; consequently, with bacterial culture results and antibiograms, definitive therapy was adjusted according to the treating physicians’ criteria.

To describe whether the inflammatory response markers were associated with pulmonary coinfection, whole blood samples for leukocyte count and serum for C-reactive protein and procalcitonin were taken on the first day of intubation. We analyzed the empirical antibiotics after taking samples from the lower respiratory tract and the changes in antibiotic management according to the results of the FA-PNEUM and cultures.

This study was conducted according to the regulations of the Universidad Pontificia Bolivariana ethics committee and the committees of each of the participating institutions. Informed consent was obtained from the patient or their legal representative for sample handling and accessing registered information in medical records.

### COVID-19 detection

Testing for SARS-CoV-2 was performed by real-time polymerase chain reaction (RT–PCR) on nasopharyngeal swabs, following WHO and/or CDC protocol, in an Allplex SARS-CoV-2 assay (Seegene, Inc. Korea) to amplify the SARS-CoV-2 E, RdRp/s, and N genes.

### Conventional culture

Respiratory samples were subjected to Gram staining and conventional semiquantitative cultures. The samples were not processed when a quality level of 0 or 1 was detected by the Murray/Washington criteria, based on the number of squamous cells and neutrophils per field [12]. Ten microliters of ETA or mini-BAL was seeded on different media: chocolate, CNA (colistin + nalidixic acid), and MacConkey agar. No sample dilution method was performed, and the samples were incubated for up to 48 h (incubated at 35 °C in an aerobic atmosphere and enriched with 5% CO_2_ for 2 days). Afterward, microorganisms that grew in significant amounts according to the guidelines of standard laboratory procedures were quantified (upon growth at or above 10^4^ CFU/mL for a mini-BAL fluid sample or 10^5^ CFU/mL for an ETA sample) and subcultured to isolate them if there were two or more microorganisms on nonselective plates. If they were negative, they were kept for further observation for up to 3 days. Cultures for viruses or atypical bacteria were not performed.

Bacterial species-level identification was conducted using VITEK® 2 identification (ID) cards. Antimicrobial susceptibility testing (AST) was performed by VITEK® 2 (bioMérieux, Marcy l’Etoile, France) following the manufacturer’s instructions. The tests were interpreted according to current Clinical & Laboratory Standards Institute (CLSI) guidelines [13]. The presence of normal respiratory microbiota and nonpulmonary pathogens such as *Candida spp*. and *Enterococcus faecalis* was considered negligible.

### BioFire® FilmArray® Pneumonia Panel (FA-PNEU)

The FA-PNEU is a syndromic panel based on multiplex PCR targeting 18 bacterial pathogens (eleven Gram-negative, four Gram-positive, and three atypical), nine viruses, and seven determinants of resistance (namely CTX-M, KPC, NDM, Oxa48-like, VIM, IMP, and MecA/C/MREJ). Viral, fungal, and atypical bacteria were reported as not detected or detected, and resistance genes were reported as positive. In the case of positive results, semiquantitative values expressed in DNA copies/mL were also reported for each pathogen detected. All steps from nucleic acid extraction to the final detection of pathogens were carried out in an automated manner. The sample swab included in the kit was used to dispense the appropriate amount of mini-BAL or ETA into the cartridge according to the manufacturer’s instructions. Briefly, approximately 200 μL of the sample was collected using a flocked swab and transferred to a sample injection vial. It was then mixed with the provided sample buffer. This solution was then loaded into the FilmArray pouch, which in turn was loaded into the FilmArray platform. The preparation of each cartridge required approximately 2 min of hands-on time, while the run time was approximately 1 h and 15 min [14, 15].

### Coinfection definition

Coinfection was defined as the identification of a respiratory tract pathogen using the FA-PNEU or microbiological cultures of respiratory samples from patients with severe COVID-19 infection.

### Data analysis

Categorical variables were expressed as frequencies and percentages. Continuous variables are presented as medians and 25^th^ and 75^th^ percentiles. Overall concordance was calculated as [(true positive + true negative/total)] × 100, positive concordance was calculated as [(true positive/total)] × 100), and Cohen's kappa coefficient with 95% confidence intervals was estimated with FA-PNEU and conventional culture results. Sensitivity, specificity, positive and negative predictive values (PPVs and NPVs), along with 95% confidence intervals, were calculated by comparing the results between conventional culture and the FA-PNEU only for bacterial pathogens present in the molecular panel. Performance was measured considering bacterial culture as the gold standard reference method. We did not perform concordance analysis or qualitative agreements for atypical bacteria or viruses. The association between the presence of coinfection and the inflammatory response was measured by the Kruskal–Wallis test. Data were entered into a Microsoft Excel database and analyzed using SPSS software version 26.0.

## Results

During the study period, 1289 patients admitted to the ICU with severe COVID-19 were screened, of which 149 met the inclusion criteria (Fig. 1). Study participant demographic characteristics are shown in Table 1. The median age was 58 years (P25 46–P75 66); 57.7% were males; the APACHE score median was 10, and the most frequent comorbidity was arterial hypertension in 46.3% of participants. In-hospital deaths occurred in 34.5% of the patients.

**Table 1:** Demographic characteristics of patients with COVID-19 pneumonia admitted to intensive care units

Characteristic (*n* = 149)	Frequency (%)
Male	86 (57.7)
Age (Me-P25–P75)	58 (46–66)
Hypertension	69 (46.3)
Diabetes	36 (24.2)
Chronic kidney disease	8 (5.4)
Rheumatologic disease	3 (2.0)
Neoplasm	3 (2.0)
Chronic obstructive pulmonary disease	2 (1.3)
HIV	2 (1.3)
Heart failure	1 (0.7)
Cirrhosis	1 (0.7)

Me, median; P25–P75, 25th and 75th percentiles; HIV, human immunodeficiency virus; %, percentage

One hundred forty-nine samples were drawn (139 by ETA and 10 by mini-BAL) from 149 patients, of which 110 samples were processed through the FA-PNEU and 149 through conventional culture. Of the 110 samples tested by both methods, a total of 27 samples (24.54%) were detected by the FA-PNEU vs. 19 samples (17.27%) detected by culture. In total, the FA-PNEU identified 40 microorganisms and cultured 23. Table 2 details the summary of pathogens detected overall and via the FA-PNEU and microbiological culturing. The overall concordance was 90.1%, and when stratified by microorganism, it was between 92.7 and 100%. The highest positive concordance was for *S. aureus* with 7.3%.

**Table 2:** Summary of total, BioFire® FilmArray® Pneumonia Panel, and microbiological culturing detections for all pathogens

Microbial target	FA-PNEU (+) microbiological culture (+)	FA-PNEU (+) microbiological culture (−)	FA-PNEU (−) microbiological culture (+)	Total (+)	FA-PNEU (+)	Microbiological culture (+)	Overall concordance	Concordant positive	Cohen's kappa coefficient (95% CI)
*Aspergillus flavus*	0	0	1	1	0	1	99.10	0.0	0
*Enterobacter cloacae complex*	2	2	0	4	4	2	98.20	1.8	65.8 (21.7; 100.9)
*Haemophilus influenzae*	0	4	0	4	4	0	96.36	0.0	0
*Klebsiella pneumoniae*	5	0	1	6	5	6	99.10	4.5	90.4 (71.9; 100.9)
*Klebsiella oxytoca*	1	0	0	1	1	1	100.00	0.9	100 (100; 100)
*Pseudomonas aeruginosa*	1	0	1	2	1	2	99.10	0.9	66.26 (4.3; 128.2)
*Streptococcus agalactiae*	0	8	0	8	8	0	92.70	0.0	0
*Staphylococcus aureus*	8	7	0	15	15	8	93.64	7.3	66.4 (43.7; 89.1)
*Streptococcus pneumoniae*	2	0	1	3	2	3	99.10	1.8	79.51 (40.4; 108.6)
Global^a^	18	9	1	28	27	19	90.10	16.4	72.7 (57.1; 88.4)

CI, confidence intervals; FA-PNEU (+), positive BioFire® FilmArray® Pneumonia Panel; FA-PNEU (−), negative BioFire® FilmArray® Pneumonia Panel

^a^Patients whose test was positive for at least one microorganism

Of the 110 samples that underwent both techniques, 18 samples were positive for both methods, 82 samples were negative for both techniques, 1 sample was culture-positive with a negative FA-PNEU result (*Aspergillus flavus*, which was assumed to be caused by contamination by medical staff), and 9 samples were FA-PNEU-positive and culture-negative (six *S. aureus*, two *Streptococcus agalactiae,* and one *Haemophilus influenzae)*. The two bacteria most frequently detected by the FA-PNEU were *S. aureus* (37.5%) and *S. agalactiae* (20%), and those detected by culture were *S. aureus* (34.78%) and *Klebsiella pneumoniae* (26.08%). Atypical bacteria were not detected by the FA-PNEU. Two samples with *rhinovirus/enterovirus* were detected by the FA-PNEU.

Among the 27 FA-PNEU-positive samples, 12 (44.44%) were polymicrobial compared with 4/19 (21.05%) culture-positive samples. The most common combinations found were *S. aureus* and *K. pneumoniae*.

According to the FA-PNEU, 40% of the detected *S. aureus* were methicillin-resistant (MRSA). In six samples, the FA-PNEU detected the MecA/C/MREJ resistance mechanism in *S. aureus*, which was not detected by conventional cultures. None of the *K. pneumoniae* isolates had CTX-M, KPC, NDM, Oxa48-like, VIM or IMP by the FA-PNEU or extended-spectrum beta-lactamases (ESBL) or carbapenemases by culture. In conventional culturing, one patient was positive for *Streptococcus pneumoniae* resistant to ceftriaxone, which was not detected by the FA-PNEU.

Table 3 depicts the qualitative agreement of eight microorganisms, that correspond to 46/110 samples of isolated microorganisms in total, detected by the FA-PNEU and culture, representing the gold standard. The positive predictive values (PPVs) were between 50 and 100% and were lower for *Enterobacter cloacae* and *S. aureus*. Negative predictive values (NPVs) were high (99.1%–100%). Regarding resistance mechanisms, MecA/C/MREJ had a specificity of 94.55% and an NPV of 100%.

**Table 3:** Comparison between BioFire® FilmArray® Pneumonia Panel and standard reference culture results (*n* = 110)

Microbial target	Sensitivity % (95% CI)	PPV % (95% CI)	Specificity % (95% CI)	NPV % (95% CI)
*Enterobacter cloacae complex*	100 (34.24, 100)	50 (15, 85)	98.15 (93.5, 99.49)	100 (96.5, 100)
*Haemophilus influenza*	–	0 (0, 48.99)	96.36 (91.02, 98.58)	100 (96.5, 100)
*Klebsiella oxytoca*	100 (20.65, 100)	100 (20.65, 100)	100 (96.6, 100)	100 (96.6, 100)
*Klebsiella pneumoniae*	83.3 (43.65, 96.99)	100 (56.55, 100)	100 (96.44, 100)	99.05 (94.8, 99.83)
*Pseudomonas aeruginosa*	50 (9.45, 90.55)	100 (20.65, 100)	100 (96.57, 100)	99.08 (94.99, 99.84)
*Streptococcus agalactiae*	–	0 (0, 32.44)	92.73 (86.3, 96.7)	100 (96.37, 100)
*Staphylococcus aureus*	100 (67.56, 100)	53.33 (30.12, 75.19)	93.14 (86.51, 96.64)	100 (96.11, 100)
*Streptococcus pneumoniae*	66.67 (20.77, 93.85)	100 (34.24, 100)	100 (96.53, 100)	99.07 (94.94, 99.84)

PPV, positive predictive value; NPV, negative predictive value; %, percentage; CI, confidence intervals

Regarding the quantitative agreement, of the microorganisms in the cultures of ETA samples with > 10^5^ CFU, 84.21% had a count of ≥ 10^5^ copies/mL in the FA-PNEU result; of the negative cultures, 40.9% had microorganisms with a count < 10^5^ copies/mL in the FA-PNEU result. The 10 samples taken by mini-BAL were negative in both cultures and the FA-PNEU.

In patients on mechanical ventilation with severe SARS-CoV-2 pneumonia, coinfection was not associated with an increase in mortality, as opposed to FA-PNEU-positive patients (44.8% vs. 32.1%; *p* = 0.219) and culture-positive individuals (40% vs. 35.2%; *p* = 0.652). The inflammatory response tests showed no significant differences between patients whose samples were positive and negative for both techniques (Table 4). Although the sample is not enough to make statistical comparisons, we can see in the table that in culture-negative patients with positive FA-PNEU results with a count of ≥ 10^5^ copies/mL, the inflammatory response is higher than in patients with positive FA-PNEU results with a count of < 10^5^ copies/mL.

**Table 4:** Results of laboratory tests of inflammatory response among patients with or without pulmonary coinfection

Combination of tests	Laboratory	Me	P25	P75	Min	Max	*n*
FA-PNEU (+) culture (+)	Procalcitonin (ng/mL)	0.13	0.09	0.53	0.05	1.66	13
	C-reactive protein (mg/dL)	16.02	8.6	21.86	1.33	36.8	17
	Leukocytes (cell numbers × 10^6^/mL)	11.05	6.92	13.5	4.45	28.3	18
FA-PNEU (−) culture (−)	Procalcitonin (ng/mL)	0.23	0.10	0.6	0.05	27.4	69
	C-reactive protein (mg/dL)	17.0	10.68	24.87	2.25	46.9	80
	Leukocytes (cell numbers × 10^6^/mL)	10.1	7.46	13.5	3.58	16.04	80
^a^FA-PNEU (+) culture (−)	Procalcitonin (ng/mL)	0.56	0.13	0.97	0.06	1.02	4
	C-reactive protein (mg/dL)	19.84	14.20	19.99	0.15	21.1	5
	Leukocytes (cell numbers × 10^6^/mL)	14.85	13.6	20.39	9.94	28.8	5
^b^FA-PNEU (+) culture (−)	Procalcitonin (ng/mL)	0.07	0.05	0.27	0.05	0.27	3
	C-reactive protein (mg/dL)	9.85	9.02	24.18	5.58	25.07	6
	Leukocytes (cell numbers × 10^6^/mL)	10.22	9.8	11.5	9.5	18.87	6

Me, Median; P25–P75, 25th and 75th percentiles; Min, minimum; Max, maximum; n, number of observations; FA-PNEU, BioFire® FilmArray® Pneumonia Panel

^a^BioFire® FilmArray® Pneumonia Panel with a count of ≥ 10^5^ copies/mL

^b^BioFire® FilmArray® Pneumonia Panel with a count of < 10^5^ copies/mL

Of the 110 cases analyzed, 61 (55.45%) received at least one dose of empirical antibiotics after LRT samples were taken. The antibiotic regimen most commonly used was ceftriaxone (45.9%), followed by cefepime (31.1%) and ampicillin/sulbactam (23%). Later, when the treating physicians knew the result of the FA-PNEU, in 58 (95.1%) of the 61 patients, the therapy was modified as follows: In 21 (91.3%) of the 23 cases in which the FA-PNEU result was positive, the therapy was changed, and in 37 (97.4%) of the 38 cases in which the FA-PNEU result was negative, the antibiotics were suspended (Table 5).

**Table 5:** Change of antibiotic management according to the results of BioFire® FilmArray® Pneumonia Panel and cultures

Condition	Test	Result	Change of antibiotic management		*n*
			Yes (%)	No (%)	
With previous antibiotic	FA-PNEUM	Positive	21 (91.3)	2 (8.7)	23
		Negative	37 (97.4)	1 (2.6)	38
		Total	58 (95.1)	3 (4.9)	61
	Culture	Positive	6 (31.6)	13 (68.4)	19
		Negative	1 (100)	0 (0)	1
		Total	7 (35)	13 (65)	20
Without previous antibiotic	FA-PNEUM	Positive	4 (100)	0 (0)	4
		Negative	0 (0)	45 (100)	45
		Total	4 (8.2)	45 (91.8)	49
	Culture	Positive	0 (0)	0 (0)	0
		Negative	0 (0)	81 (100)	81
		Total	0 (0)	81 (100)	81

n, number of observations; FA-PNEU, BioFire® FilmArray® Pneumonia Panel; %, percentage

Forty-nine (44.55%) of the 110 patients did not receive empiric antibiotics after LRT samples were taken. Of these, the FA-PNEU result was negative in 45 cases, and no antibiotics were started; in the other 4 cases where the FA-PNEU result was positive, antibiotics were started (Table 5). The antibiotics that were most used in this phase of treatment after knowing the result of the FA-PNEU were oxacillin (33.3%) and linezolid (23.8%). Finally, in the patients who had antibiotics and positive cultures, the treatment was changed in 6 (31.6%) of the 19 patients; the most frequent change was de-escalation therapy to ciprofloxacin (50% of cases). In one case with a negative culture, the treatment was discontinued.

## Discussion

Our article has several take-home messages for the management of patients with COVID-19 pneumonia admitted to the ICU: First, approximately a quarter of patients with COVID-19 pneumonia admitted to the ICU have bacterial coinfection; second, a negative FA-PNEU result prevents the inappropriate empirical use of antibiotics in these patients as a stewardship strategy for COVID-19; and third, the overall concordance between FA-PNEU and culture was 90.1%, and it was between 92.7% and 100% when stratified by microorganisms.

Bacterial coinfection in critically ill COVID-19 patients occurred in 24.54% and 17.27% of patients tested by the FA-PNEU and conventional cultures, respectively. In the most recent meta-analysis [16] on the identification of bacterial coinfections by the FA-PNEU in ICU-hospitalized COVID-19 patients, four of the seven studies reported on the timing of specimen collection within the first 48 h of ICU admission. In total, 221 patients were included, and the pooled incidence of coinfections detected by the FA-PNEU was 33% (95% CI 0.25–0.41) and 18% by conventional cultures (95% CI 0.02–0.45) [7–10]; the incidence is higher than that reported in inpatient services, which ranges between 3.5 and 8% [4, 17]. The largest study by Kolenda et al.[8] included 99 patients admitted to 3 ICUs in France, and the samples were taken in the absence of mechanical ventilation or within 48 h after mechanical ventilation was initiated; cultures identified 17 bacteria in 15 of 99 samples (15.1%).

The two most frequently detected bacteria were *S. aureus* (37.5%) and *S. agalactiae* (20%) by the FA-PNEU and *S. aureus* (34.78%) and *K. pneumoniae* (26.08%) by culture. Verroken et al. [10] reported the results of 32 respiratory samples from 41 COVID-19 patients in the ICU; the FA-PNEU identified 13/32 (40.6%) patients with a bacterial coinfection, where *S. aureus* (38.46–60% methicillin-sensitive), *H. influenzae* (23.07%), and *Moraxella catarrhalis* (15.38%) were the main pathogens identified. Kreitmann et al. [9] documented bacterial coinfection in 13 of 47 subjects (27.7%) from samples taken within 24 h of tracheal intubation, with three bacterial species representing ≥ 90% of those identified: *S. aureus* (69.2%, all methicillin-sensitive), *H. influenzae* (38.5%), and *S. pneumoniae* (23.1%). Kolenda et al. analyzed 99 patients with respiratory samples taken in the absence of mechanical ventilation or during their first 48 h; conventional cultures detected bacterial coinfection in 15%, with *S. aureus* (46.6%, all methicillin-sensitive), *H. influenzae* (26.66%), and *S. pneumoniae* (13.33%) being the most prevalent pathogens.

When comparing the aforementioned studies with our work, three aspects are worth highlighting: First, in all the studies, including ours, *S. aureus* was the most prevalent microorganism; second, in the present study, methicillin resistance was higher (40% of the FA-PNEU isolates had MecA/C/MREJ, while in the cultures, no methicillin resistance was found); and last, unlike other studies, *K. pneumoniae* was the second most prevalent microorganism in the current study by culture, with no ESBL or KPC resistance mechanisms.

Regarding the qualitative agreements between the FA-PNEU and conventional cultures, in our study, we found that the PPV was between 50 and 100% and lower for *E. cloacae* and *S. aureus*; the NPV was high (between 99.1% and 100%). Caméléna et al. [7] demonstrated that the results of the FA-PNEU are consistent (sensitivity 95%, specificity 99%, PPV 82%, and NPV 100%) with those of conventional culturing for bacterial pathogens of 96 samples from 43 intubated patients with suspected bacterial coinfection or superinfection; *S. aureus*, as opposed to our study, did have a good PPV (91%). Kolenda et al. [8] reported a FA-PNEU sensitivity of 100%, since all isolated bacteria in cultures were also detected using the FA-PNEU, with a specificity of 98.7%; the lowest specificity was for *H. influenzae* (< 88.4%), and the specificity for *S. aureus* was 93.5%.

In our study, tests for *S. aureus* had a sensitivity of 100%, a PPV of 53.3%, a specificity of 93.1%, and an NPV of 100%, since 6 of the 9 patients with FA-PNEU-positive and culture-negative microorganisms were positive for *S. aureus*. Moreover, in 6 FA-PNEU samples, the MecA/C/MREJ resistance mechanism was detected and not identified by conventional cultures. Fontana et al. [18] used the FA-PNEU to assess coinfection in 152 respiratory specimens from COVID-19 inpatients; 23 of them required assisted ventilation in the ICU. The most representative species was *S. aureus* in both BAL (21; 16 mecA positive) and sputum (27; 14 mecA positive), with the majority being mecA positive (30/44, 62%). Although most of the patients were not in the ICU, their results are consistent with our findings.

Concerning the quantitative agreement, in our study, microorganisms in cultures of ETA samples with > 10^5^ CFU, 84.21% had a count of ≥ 10^5^ copies/mL in FA-PNEU testing; of the culture-negative samples, 40.9% had microorganisms with a count < 10^5^ copies/mL in FA-PNEU testing. In the study of Kolenda et al. [8] among 16 bacteria reported in cultures, 15 (93.8%) showed ≥ 10^6^ copies/mL using the FA-PNEU; in contrast, among 26 bacteria detected using the FA-PNEU yet culture-negative, 20 (76.9%) had ≤ 10^5^ copies/mL using the FA-PNEU. We can conclude that most positive samples in the FA-PNEU results, with negative cultures, have low DNA copies/mL. These findings raise the following questions: Is it possible that in these cases, it is not strictly a coinfection and rather a contamination by the endogenous flora? What is the clinical impact of this finding? While culturing remains the gold standard in the diagnosis of bacterial respiratory tract infections, it may be difficult to accurately recover all pathogens in clinical samples, as the organisms are in a complex matrix. In addition, culture results would be more affected by the host immune response and prior antibiotic usage. Antimicrobial therapy can impact bacterial growth, leading to negative cultures but to persistent positive FA-PNEU results, which is not able to distinguish dead from viable bacteria. For this reason, we excluded patients who, at the time of respiratory sample collection, had received any dose of empiric antimicrobial therapy. Distinguishing colonizing organisms from pathogens remains a challenge because levels of bacteria below the culture threshold can provide positive results in the FA-PNEU. However, previous studies have described that the bacterial burden could be overestimated by FilmArray compared to culturing [19, 20].

In future studies, we will try to give some answers by comparing the results of the cultures and FA-PNEU with samples from the lung microbiome through the extraction of DNA and RNA for sequencing of the same samples for metagenomics and metatranscriptomics, which will allow us to determine the functional profiles of the virulence and resistance genes of microorganisms and differentiate expressed human genes. These strategies that examine the host response provide opportunities to rethink what defines true pneumonia and lung coinfection, so some propose a reconceptualized view of pneumonia, in which the development of pneumonia is believed to result from disruption of the complex homeostasis of a microbial ecosystem interacting with multiple complex growth conditions [21].

We did not observe differences between inflammatory biomarker levels and pulmonary coinfection defined by the samples that were positive and negative for both techniques, the FA-PNEU and culturing. However, although the sample was not enough to make statistical comparisons, we found that in culture-negative patients with positive FA-PNEU results with a count of < 10^5^ copies/mL, the inflammatory response was lower than that in patients with positive FA-PNEU results with a count of > 10^5^ copies/mL, which could suggest that these patients have more colonization than infection. There are few studies evaluating whether acute phase reactants are useful as predictors of coinfection in patients with COVID-19. Bolker et al. [22] found that the risk factors for respiratory bacterial coinfection upon hospital admission were nursing home stay, severe COVID-19, and leukocytosis; the other inflammatory markers within 72 h of admission (procalcitonin, CRP, IL-6, and ferritin) were not predictors. Mason et al. [23], in a retrospective cohort study of patients with community-acquired pneumonia and patients with COVID-19, proposed that in COVID-19, the absence of both leukocytosis and an antibiotic-related decrease in C-reactive protein can exclude bacterial coinfection.

We did not find that coinfection by culture or by the FA-PNEU was associated with an increase in mortality. Another Latin American study conducted by Soto et al. evaluated ninety-three hospitalized patients with a diagnosis of COVID-19 who were analyzed with the FA-PNEU. Coinfection was evidenced in 38 (40.86%) cases, and no association with mortality was found (OR 1.63; 95% CI 0.45–5.82) [24].

Based on only a few studies without defined information on sampling strategies, a bacterial or fungal coinfection rate of 8% in COVID-19 patients was estimated, but 72% of all these reported COVID-19 patients received (empiric broad-spectrum) antibiotic therapy [17]. In a study from Germany, of 135 analyzed cases, most patients received antimicrobial therapy within 24 h of admission (n = 109, 80.7%), and 46.0% of severely ill patients admitted to the ICU developed coinfections [25]. In our data, of the 110 cases analyzed, 61 (55.45%) received at least one dose of empirical antibiotics after LRT sample collection, and when the treating physicians knew the result of the FA-PNEU, the therapy was modified in 58 (95.1%) of the 61 patients; furthermore, in 37 (97.4%) of the 38 cases in which the FA-PNEU result was negative, the antibiotics were suspended. The high NPV of the FA-PNEU allows us to quickly suspend or not start antibiotics as a strategy for improving antimicrobial stewardship in COVID-19.

A limitation of our study is that the samples were collected either by endotracheal aspirate or by mini-BAL and not through BAL. However, we did this for several reasons: First, the latest US guidelines recommend noninvasive sampling (endotracheal aspiration) with semiquantitative cultures to diagnose VAP and non-VAP; there is no evidence that invasive microbiological sampling with quantitative cultures improves clinical outcomes compared with noninvasive sampling with either quantitative or semiquantitative cultures. Second, noninvasive sampling can be performed more rapidly than invasive sampling, with fewer complications and resources; it is our usual practice due to the lack of availability of pulmonary professionals 24 h a day, and this applies even more during the COVID-19 pandemic. Third, the ETA samples were collected properly, and the respiratory samples were not processed when a quality level of 0 or 1 was detected by the Murray/Washington criteria, based on the number of squamous cells and neutrophils per field. Fourth, only results with growth ≥ 10^4^ CFU/mL for a mini-BAL fluid sample or ≥ 10^5^ CFU/mL for an ETA sample were considered; we did not take into account growth of < 10^5^ for an ETA sample to avoid overestimating the prevalence of coinfection. Fifth, the BioFire® FilmArray® Pneumonia Panel technique has been validated for ETA samples [26]

There are several strengths in this study. First, the high NPV of the FA-PNEU was demonstrated; therefore, we can conclude that if we find a negative result, bacterial coinfection is practically excluded. Novy et al. proposed an algorithm for the rational use of the FA-PNEU in critically ill ventilated COVID-19 patients; this would allow 65.6% of antibiotic sparing for bacterial coinfection and better adequacy of empirical antibiotic therapy [27]. Second, our investigation is one of the studies with the largest number of included patients with the aim of assessing the diagnostic concordance of the FA-PNEU with culturing in subjects with COVID-19 pneumonia admitted to ICUs. Third, this is the first Latin American study with this purpose; most of the studies have been carried out in Europe. Finally, we analyzed whether inflammatory response markers were associated with pulmonary coinfection and the changes in antibiotic management according to the results of the FA-PNEUM and cultures.

## Conclusions

Bacterial coinfection in critically ill COVID-19 patients was present in 24.54% of samples tested by the FA-PNEU and in 17.27% of samples tested by conventional culture. The most frequently isolated microorganism was *S. aureus.* Concerning qualitative agreements between the FA-PNEU and conventional cultures, we found that the PPV ranged between 50 and 100% and was lower for *E. cloacae* and *S. aureus*, with a high NPV (between 99.1 and 100%). The overall concordance was 90.1%, and it was between 92.7 and 100% when stratified by microorganisms.

## Data Availability

All data generated or analyzed during this study are included in this published article. The datasets generated or analyzed during the current study are available from the corresponding author on reasonable request.

## Abbreviations

FA-PNEU: BioFire® FilmArray® Pneumonia plus Panel
RT-PCR: Real-time polymerase chain reaction
ICU: Intensive care unit
SRAS-CoV-2: Severe acute respiratory syndrome coronavirus 2
PPV: Positive predictive values
NPV: Negative predictive values
COVID-19: Infectious disease caused by a coronavirus-2019
LRTI: Lower respiratory tract infection
ETA: Endotracheal aspirate
mini-BAL: Mini-bronchoalveolar lavage fluid
DNA: Deoxyribonucleic acid
RNA: Ribonucleic acid
AST: Antimicrobial susceptibility testing
CLSI: Clinical and Laboratory Standards Institute
MRSA: *Staphylococcus aureus m* *ethicillin-resistant*
ESBL: Extended-spectrum beta-lactams
